# Towards Mobile Gait Analysis: Concurrent Validity and Test-Retest Reliability of an Inertial Measurement System for the Assessment of Spatio-Temporal Gait Parameters

**DOI:** 10.3390/s17071522

**Published:** 2017-06-28

**Authors:** Felix Kluge, Heiko Gaßner, Julius Hannink, Cristian Pasluosta, Jochen Klucken, Björn M. Eskofier

**Affiliations:** 1 Digital Sports Group, Pattern Recognition Laboratory, Department of Computer Science, Friedrich-Alexander University Erlangen-Nürnberg (FAU), 91058 Erlangen, Germany; julius.hannink@fau.de (J.H.); cristian.pasluosta@imtek.uni-freiburg.de (C.P.); bjoern.eskofier@fau.de (B.M.E.); 2 Molecular Neurology, University Hospital Erlangen, Friedrich-Alexander University Erlangen-Nürnberg (FAU), 91054 Erlangen, Germany; heiko.gassner@uk-erlangen.de (H.G.); jochen.klucken@uk-erlangen.de (J.K.); 3 Laboratory for Biomedical Microtechnology, Department of Microsystems Engineering, University of Freiburg, 79110 Freiburg, Germany

**Keywords:** walking, stride parameters, ambulatory motion tracking, human gait, movement analysis, inertial measurement unit, sensors, wearable sensors, accelerometer, gyroscope

## Abstract

The purpose of this study was to assess the concurrent validity and test–retest reliability of a sensor-based gait analysis system. Eleven healthy subjects and four Parkinson’s disease (PD) patients were asked to complete gait tasks whilst wearing two inertial measurement units at their feet. The extracted spatio-temporal parameters of 1166 strides were compared to those extracted from a reference camera-based motion capture system concerning concurrent validity. Test–retest reliability was assessed for five healthy subjects at three different days in a two week period. The two systems were highly correlated for all gait parameters (r>0.93). The bias for stride time was 0±16 ms and for stride length was 1.4±6.7 cm. No systematic range dependent errors were observed and no significant changes existed between healthy subjects and PD patients. Test-retest reliability was excellent for all parameters (intraclass correlation (ICC) > 0.81) except for gait velocity (ICC > 0.55). The sensor-based system was able to accurately capture spatio-temporal gait parameters as compared to the reference camera-based system for normal and impaired gait. The system’s high retest reliability renders the use in recurrent clinical measurements and in long-term applications feasible.

## 1. Introduction

Parkinson’s disease (PD) is the most frequent neuro-degenerative disorder with a high prevalence [1]. It is characterized by movement impairments with the cardinal symptoms tremor, bradykinesia, rigidity, and loss of postural reflexes as well as secondary gait symptoms such as shuffling gait and freezing [2]. To clinically assess motor symptoms, the patient’s gait impairment is observed and rated on a four-point scale as a subitem of the Unified Parkinson’s Disease Rating Scale (UPDRS-III) [3]. However, it has been demonstrated that subjectively performed observational gait assessment shows only moderate reliability and validity [4,5], and hence more objective measures are needed in order to support diagnosis, assessment of treatments, and therapeutic decision making.

Gold standard motion analysis systems such as marker-based infrared cinematography are useful for obtaining accurate estimates of gait kinematics and gait parameters for clinical diagnosis. However, they are stationary, expensive and require manual post processing, limiting their feasibility in clinical practice [6]. Clinical or laboratory environments may also cause deviations from everyday walking patterns and suffer from potential patient-doctor expectancy effects [7]. Additionally, clinical visits are only snapshots of the patients history. Responses to treatment such as medication are not necessarily captured in singular clinical visits, and more frequent updates on health statuses might be desirable [8].

Mobile gait analysis systems are a promising approach to assist clinical decision making in the hospital by supplying objective gait measures with less expertise required than for motion capture systems [6,9]. Furthermore, they can be applied to monitor long-term gait changes and responses to treatments in free living environments (e.g., in home monitoring settings) [10] without the need for regular appointments and potential patient–doctor expectancy effects might be reduced.

Separating disease and treatment effects detected by the sensor system from measurement errors of the system is essential for clinical applications. It is therefore important to evaluate the measurement system’s validity as compared to external reference systems (i.e., concurrent validity). In the context of recurrent clinical measurements, test–retest reliability of the spatio-temporal gait parameters is important and a prerequisite if the measurement system is employed to monitor longitudinal treatment effects over several weeks or if the measurement system is transferred to unsupervised home monitoring applications.

Wearable inertial measurement units have increasingly been used in gait analysis applications with their validity or reliability usually assessed in healthy subjects [11,12,13]. Hamacher et al. presented a mobile system exhibiting a high reliability, which was based on sensors attached to the forefeet [11]. They used a sensor recalibration technique in order to improve retest reliability of gait parameters. Donath et al. assessed the reliability of a sensor attached to the lateral aspects of the shoe [12], but only assessed treadmill and not overground walking. Orlowski et al. employed sensors laterally attached to the shanks above the ankle to derive gait parameters [13]. Gait event detection was based on angular shank velocity, which might be problematic for pathological gait, as angular velocities are only indirect measures of those gait events [14]. The mobile gait analysis systems above were thus based on different sensor setups and gait parameter extraction algorithms. Validity and reliability was, however, only assessed with healthy subject populations.

Mobile gait analysis system are, however, often designed for spatio-temporal gait parameter extraction in clinical applications such as disease assessment and treatment evaluation [10,15,16,17]. Patient populations that exhibit specific gait disorders should therefore also be assessed regarding validity and reliability. Papi and colleagues, for example, investigated the validity of wearable sensors in a rehabilitating knee osteoarthritis population with three sensors attached to the lower body (knee, thigh, waist), but no sensor was attached to the feet [18]. Kobsar and colleagues investigated different attitude correction methods for sensors’ positions at the back, thigh, shank, and foot concerning reliability with knee osteoarthritis patients and characterized acceleration waveforms, which can potentially replace biomechanical measures such as spatio-temporal gait parameters. However, they do not provide a direct clinical interpretation of clinical parameters [19]. Kitawaga and colleagues employed foot-worn sensors to calculate foot trajectories and associated gait parameters by direct integration and achieved a mean accuracy of 2.0 ± 5.0 cm for stride length [20].

In this study, we present the evaluation of a new sensor-based gait analysis system that employs lightweight sensors worn only on the feet for the extraction of clinically interpretable spatio-temporal gait parameters using state-of-the-art algorithms. The main aim of our study was to assess the concurrent validity of this mobile system against an external camera-based system for a healthy and PD patient population and to assess the test–retest reliability over three measurement sessions with healthy subjects. We hypothesized that the gait parameters obtained present an accurate estimate of the subjects’ gait patterns that render the use in clinical applications and long-term monitoring studies feasible. We used a markerless motion capture system as reference system, which could potentially be used complementary to enhance gait assessment in future semi-supervised scenarios.

## 2. Materials and Methods

### 2.1. Subjects

Eleven healthy subjects and four patients suffering from PD volunteered for this study. The healthy subjects reported no orthopedic or neurological disorders, acute pain or other complaints that might have affected gait. The patients were recruited from the Movement Disorders outpatient clinic of the Department of Molecular Neurology at the University Hospital Erlangen, Germany. PD was defined according to the Guidelines of the German Association for Neurology (DGN), which are similar to the UK PD Society Brain Bank criteria [21]. All subjects gave their informed consent for inclusion before they participated in the study. The study was conducted in accordance with the Declaration of Helsinki, and the protocol was approved by the local ethics committee of the University Hospital Erlangen (Re.-No. 4208).

Prior to data acquisition, clinical ratings of the patients with PD were acquired by a Movement Disorder specialist. The motor score of the UPDRS-III and the Hoehn and Yahr disease staging were used to assess disease severity and clinical symptoms [3,22]. The population characteristics are shown in Table 1.

### 2.2. Study Protocol

A battery of different short distance walking tests that provide clinically useful measures in a population of patients with PD [23] were performed as previously described [24]. For the following evaluation, only data from the 4 × 10 m walking test at different speeds was considered, as this test provided straight walking distances. This test consists of walking four times a straight 10 m distance with turning movements in between. The subjects performed the test at three different self-selected walking speeds (fixed order: normal, slow, fast) to cover a high range of different walking speeds.

For the test–retest evaluation, we performed this protocol with five of the healthy subjects at three different days during a two week measurement period. The data acquisition was performed by the same examiner in all measurement sessions. The data of this study is accessible for collaborative research [25].

### 2.3. Measurement Setup

**Figure 1:**
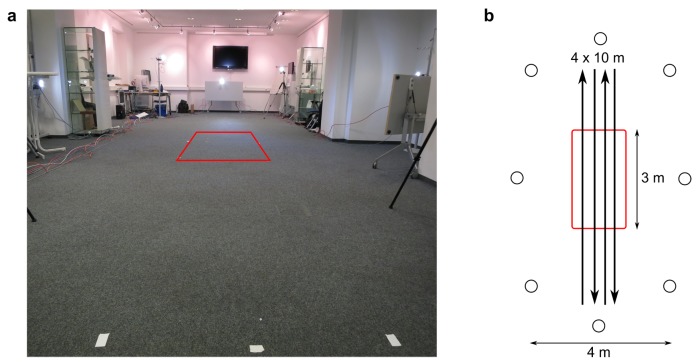
(**a**) measurement setup; (**b**) placement of cameras around the 10 m walkway. The red box indicates the volume in which full body markerless tracking using all eight cameras could be performed. The 4 × 10 m walk is schematically shown.

The measurements took place in a laboratory environment within the METEAN center (joint venture of the Fraunhofer Institute for Integrated Circuits and the University Hospital Erlangen, Germany). The eight cameras of the camera-based reference system were positioned around a 10 m walkway (Figure 1). Due to the limited field of view of the cameras, full body motion was visible in all eight cameras only in the middle of the acquisition volume along 3 m of the 10 m walkway as indicated by the red box in Figure 1. Motion tracking data based on less than eight cameras were neglected in the subsequent analysis to ensure good tracking results.

By using only the strides from the middle section of the measurement volume, we also assured that the subjects were walking at steady speed in all assessments and that no turnings, initiation or stopping strides were included in the further analysis.

### 2.4. Sensor-Based Gait Analysis

**Figure 2:**
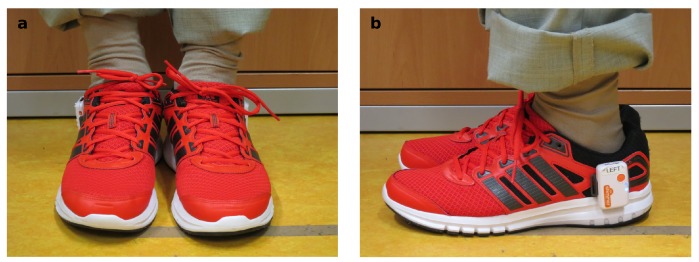
Attachment of the sensors to the shoes. (**a**) frontal view; (**b**) lateral view.

We used an inertial measurement setup consisting of two Shimmer3 sensors (Shimmer, Dublin, Ireland) [26], which contained a three-axis gyroscope (range: ±500${}^{\circ }s^{-1}$) and a three-axis accelerometer (range: ±8 g) sampling at a rate of 102.4 Hz. The data was transferred via Bluetooth to a mobile device for storage. The sensors were attached laterally to each shoe below the ankle joint by using rigid sensor mounts (Figure 2). All subjects wore the same shoe model (Adidas Duramo 6, Herzogenaurach, Germany) for practical reasons and to restrict gait differences due to differing footwear [27]. This sensor setup has previously been used for the detection of motor impairment in PD patients [28].

The calculation of spatio-temporal parameters of our system was based on the following steps: stride segmentation, followed by the determination of gait events to determine temporal parameters and foot trajectory calculation to derive spatial parameters. The processing of the sensor data was performed in Matlab (R2016b, MathWorks Inc., Natick, MA, USA).

First, a sensor calibration of the raw sensor signals to physical units was accomplished using a calibration procedure described by Ferraris and colleagues [29]. Due to the mirrored mounting of the sensors at the lateral side of the shoes, the axes had to be aligned in order to process the signals of the left and right foot analogously.

From the continuous inertial data stream, single strides were detected using the multi-dimensional sub sequence dynamic time warping approach (msDTW) as described by Barth and colleagues [30]. The method uses a template based approach to nonlinearly match time series of different length to a pre-defined template. In the application described by Barth et al. [30], this allows the identification of single strides within an inertial data stream. Their approach involves computing a distance function based on accumulated costs between the gait signal and the template that identifies suitable starting positions of template-matches. From these starting positions, optimal segments are then found based on the previously computed costs. The DTW threshold is variable and has been set to 35 in our study, which has previously been identified to be suitable for stride segmentation [30]. It has been shown, for the sensor setup used in this study, that the gyroscope information of the sagittal and rotational plane yielded the best segmentation results regarding recall and precision in healthy elderly subjects, patients with PD, and geriatric patients in standardized gait tests and free walks [30]. The template in this study was based on 25 healthy elderly subjects and 681 individual strides.

For each individual stride, the gait events heel strike (HS), toe off (TO) and mid stance (MS) were detected. HS corresponded to the maximal deceleration of the sensor in walking direction at ground contact. TO was defined as the change from plantar flexion to dorsal extension of the foot, which was equivalent to a zero crossing in the corresponding gyroscope signal. MS was the time point with lowest energy in all gyroscope axes and corresponded to the foot resting flat on the ground. More details can be found in the work of Rampp and colleagues [31].

For the estimation of the foot orientation, the Euston complementary filter was used [32]. In contrast to simpler orientation estimation schemes that solely rely on integration of gyroscope data, the Euston filter additionally uses the acceleration signal to gain orientation clues and incorporates them in the estimation process. Based on the estimated orientation over each stride, the measurements from the local frame of measurement, which was fixed to and rotated with the shoe, were then transformed to a global coordinate frame, which was stationary and given by the initial position and orientation of the shoe. In this global frame, gravity removal was achieved by subtracting the constant, downward gravity component from the measured signal.

The gravity-free acceleration signal was then used for the estimation of the foot’s trajectory by double integration. Because direct integration of the acceleration signals shows drift effects, the following algorithm for drift correction was used. Zok and colleagues [33] proposed a combination of the direct (trapezoidal) integration of the acceleration signal with an integration of the reverse acceleration signal with known boundary conditions (zero-velocity assumption) and a subsequent fusion of both signals by a sigmoid shaped weighting function to obtain a drift free velocity signal. From the drift free velocity signal, the trajectory was then obtained by direct integration. We then calculated the spatio-temporal parameters stride time, stance time, swing time, stride length and gait velocity from the gait events HS and TO and from the foot trajectories.

### 2.5. Camera-Based Gait Analysis

The reference video data was acquired using an optical markerless motion capture system (Simi Reality Motion Systems, Unterschleißheim, Germany) with eight 0.3 megapixel (MP) color cameras (Basler scA640-120gc cameras, resolution of 658 × 492 pixels, Ahrensburg, Germany). The sampling rate was 100 Hz. Calibration of the measurement volume was performed to define a global coordinate system and to correct for camera distortion. Calibration and subsequent data acquisition was performed using the software Simi Motion (version 9.2.1, Simi Reality Motion Systems, Unterschleißheim, Germany). We synchronized the camera with the sensor system by clapping the two inertial sensors together in front of one camera before and after each measurement. We then aligned the data sets based on those initial and final synchronization events. We used the markerless motion tracking capabilities of the software Simi Shape 3D (version 2.2.1, Simi Reality Motion Systems, Unterschleißheim, Germany) for the tracking of the subjects’ body segments. The process was based on silhouette motion tracking and was composed of the following steps. By subtracting an initially acquired empty background image of the captured volume from the motion images, the 2D silhouettes of the subject were extracted for each single camera. Then, a 3D silhouette was calculated by combining multiple camera views. A biomechanical model consisting of 16 segments (pelvis, torso, neck, head, upper arm, forearm, hand, thigh, shank, and foot) was fitted to the 3D silhouette for each consecutive frame. To assure good silhouette extraction, we used the same lighting conditions (shading against sunlight and constant artificial lighting), and the subjects were asked to wear clothing with a good contrast to the laboratory background.

In order to calculate spatio-temporal gait parameters, we first labeled the gait events heel strike (HS), toe off (TO) and heel off (HO) in the raw video stream using the data acquisition software. These events subdivide the gait cycle into sub phases [34]. HS was characterized by the heel touching the ground as indicated by a soft heel deformation of the shoe. TO was defined as the last frame at which the toe was still in contact with the ground. HO was the first frame in which upward movement of the heel could be detected visually in the video stream. The same examiner performed manual frame by frame labeling of all measurement trials using the views from all eight cameras. The reference data was thus based on a semi-automatic gait analysis system composed of manual gait event detection and automatic trajectory calculation.

The trajectories and gait events were exported and further processed in Matlab. The events were used to directly calculate stride, stance and swing time. The trajectory of the proximal joint of the foot as biomechanically modeled and the stride time were then used to calculate stride length and velocity to obtain the same gait parameters as described for the sensor-based system.

### 2.6. Statistical Analysis

A total of 1166 strides was used for the subsequent statistical analysis of concurrent validity and test–retest reliability, which was performed in R version 3.4.0 (R Development Core Team, Vienna, Austria) [35]. From healthy subjects, 1037 strides were used for the evaluation, while the remaining 129 strides originated from patients. We assessed concurrent validity of all above mentioned gait parameters by calculating Pearson’s correlation, bias (mean difference), absolute error and the relative absolute error as agreement measures between the two systems. We used all single strides from all speeds in the validity analysis to cover a large range of gait parameters.

Correlation alone is not a sufficient measure of agreement, as it measures only the strength of relation between two variables. A perfect correlation would be obtained for any linear relationship between both systems. Thus, any scaling of the measurements would not change the correlation, but would strongly affect the agreement. Bland–Altman diagrams are more specific, since they present residual like plots of the differences of observed pairs of system readings against the mean values. They therefore also yield information about magnitude dependent systematic errors [36]. Additionally, Bland–Altman diagrams visualize the mean of the difference (bias) as well as the 95% confidence interval of the bias (i.e., limits of agreement).

Agreement between both systems was also assessed for healthy and patient group separately to evaluate whether mildly affected gait would affect the system’s accuracy. Differences between both populations were assessed using independent *t*-tests with a priori significance levels α of 0.05, assuming normality and homogeneity of variance.

To determine test–retest reliability, intraclass correlation (ICC) was calculated [37,38]. The ICC assesses the ratio of the intraclass variation in the regarded parameter to the between-class variation due to repeated measurements. The basis of calculating ICCs are thus analysis of variance models that include as variation terms the individual deviation from the overall population mean (subject factor), systematic test errors in the retest measurements (test factor) as well as random measurement errors. We chose a two-way model, as we expected the subject and test effect to be significant in our study and used a two-way random effect model to calculate the reliability of a single measurement ICC(2,1) and the reliability of the average measurement ICC(2,*k*) (with k=3 repeated measurements) as evaluation metrics of the test–retest assessment. We evaluated the ICC concerning the mean gait parameters (per subject and leg) using all strides of each 4 × 10 m gait test, as the average parameters per test are usually of clinical interest. We therefore included # legs·# subjects=10 samples in the ICC calculation for each of the three measurement dates. ICC values below 0.40 were considered to be poor, between 0.40 and 0.59 to be fair, between 0.60 and 0.74 to be good, and above 0.75 to be excellent [39].

## 3. Results

### 3.1. Concurrent Validity

Mean (±SD) values of the investigated spatio-temporal gait parameters together with agreement measures are given in Table 2. High correlations (r>0.93) with low errors were observed for all gait parameters. While no bias was observed for the stride time, the stance time was slightly overestimated and the swing time underestimated, respectively, by 37 ms. The stride length was underestimated by 1.4 cm and the velocity was underestimated by 1.2 cm/s. The absolute relative error of the sensor-based gait parameters was below 9% for all parameters. The best agreement was present for stride time (1.1%) and the worst for swing time (8.3%). The results of the parameters involving spatial information (stride length and velocity) showed an error of less than 4%.

**Figure 3:**
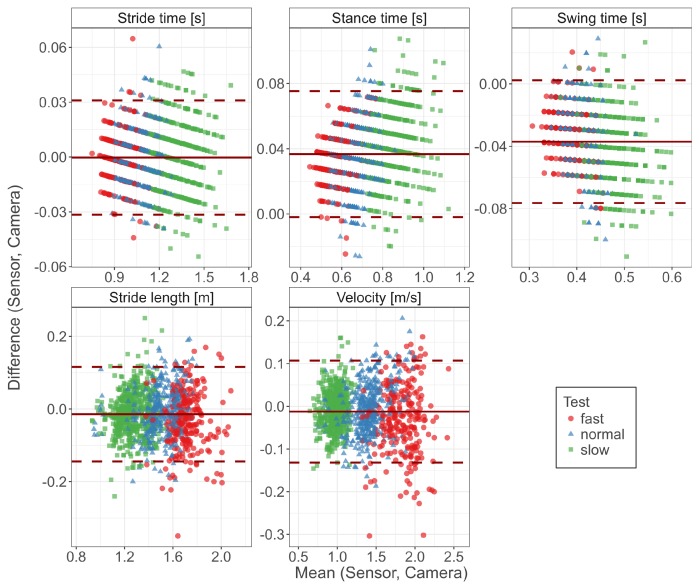
Bland–Altman diagrams of gait parameters show the difference versus the mean of both systems for all single strides. The solid line indicates the bias and the dashed lines the limits of agreement (95% confidence interval of the bias). Highlighted by colors are the three different test speeds (normal, slow, fast).

Good agreement of the sensor-based system compared to the reference system was observed for all gait parameters (Figure 3). Walking speed did not influence the bias. Only velocity showed slightly higher errors at higher walking speeds. The regular patterns for stance time, swing time and stride time arise from the temporal discretization (camera system: 100 Hz; sensor system: 102.4 Hz), which limits the accuracy of temporal measurement to around 10 ms.

Table 3 compares the agreement as grouped by the study participants being either healthy subjects or patients with PD. All gait parameters between both populations were significantly different (p<0.001 for both systems). The agreement measures were similar for both populations. The bias was significantly different for stance time (p=0.005) and stride length (p=0.018) and not significant for stride time (p=0.072), swing time (p=0.389) and velocity (p=0.055).

**Figure 4:**
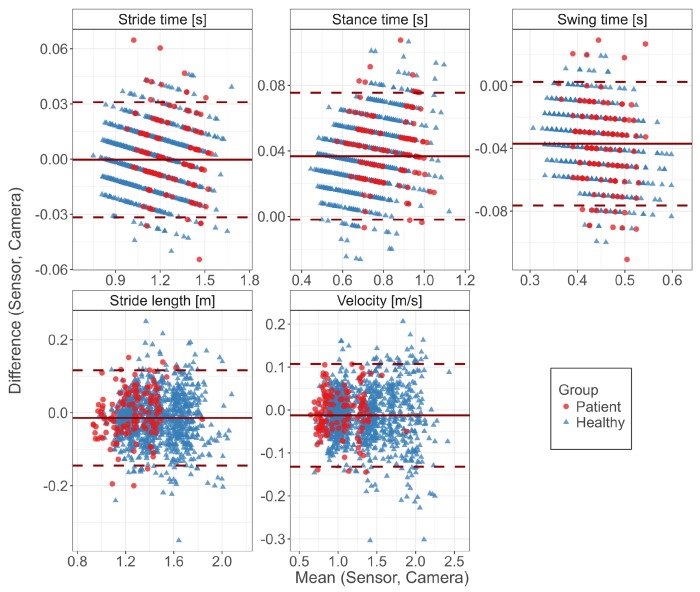
Bland–Altman diagrams of gait parameters show the difference versus the mean of both systems for all single strides. The solid line indicates the bias and the dashed lines the limits of agreement (95% confidence interval of the bias). Highlighted by colors are healthy subjects and the patient group.

Figure 4 visualizes the agreement between both systems regarding both populations in Bland–Altman diagrams. While the patients cover higher stride times, the bias and distribution of the errors were not affected by the PD condition. This is also reflected by the corresponding agreement measures (Table 3).

### 3.2. Retest Reliability

The single measurement intraclass correlation ICC (2,1) was excellent for stride time (>0.89), stance time (>0.87), swing time (>0.81) and the stride length (>0.81) in both measurement systems (Table 4). Gait velocity showed good reliability in the fast and normal walking conditions and fair reliability for slow walking. When considering the use of multiple measurements (ICC(2,*k*)), all gait parameters reach an excellent reliability above 0.79. Both measurement systems exhibit similar high ICCs for all gait parameters, indicating that both systems can be employed equally well for the determination of spatio-temporal gait parameters.

## 4. Discussion

The present study provides evidence that the employed sensor-based gait analysis system is a promising tool for the assessment of spatio-temporal gait parameters. The results suggest very good agreement between the sensor-based and the camera-based gait analysis system. Low errors were observed for stride time, stride length and velocity, while the separation of the stance and swing phases showed slightly worse results. The spatio-temporal gait parameters measured in this study are in agreement with the expected parameters determined in literature concerning healthy adults and patients with PD [40,41], indicating that reasonable gait parameter ranges have been measured. The bias of the gait parameters was not speed dependent. Only the variance of the bias increased slightly at higher speeds. Additionally, no systematic magnitude dependent errors were obvious from the investigation via Bland–Altman diagrams.

The parameters involving spatial information are dependent on accurate foot orientation estimation and double integration. As the accuracy of the stride length showed very good results, the employed methods (Euston complementary filter for orientation estimation [32] and direct and reverse integration for the velocity estimation [33]) are promising algorithms for trajectory estimation and gait parameter extraction. We obtained with our system similar results compared to other sensor-based systems for the assessment of spatio-temporal parameters [18,20,42]. Papi and colleagues used treadmill walking [18], which could, however, affect the subjects’ gait patterns [43]. Whether our sensor system and the associated algorithms are independent of overground or treadmill walking should be assessed in a future study.

The highest errors occurred for stance and swing time. Those phases are separated by the toe-off gait event, which was defined as the change from plantar flexion to dorsal extension of the foot. However, as the foot is not a rigid body segment, this assumption may not hold for all strides and might vary inter-individually, thus potentially leading to a less accurate detection of the toe-off event.

The sensor system’s accuracy did not differ between healthy subjects and patients. Besides disease, age was a major discriminating factor between the two populations, which should be considered when interpreting the gait parameters obtained. A major limitation of this study is that only four PD patients were included (UPDRS-III scores ranged between 14 and 26). For severely impaired gait, stride segmentation might work less reliably. Deviations from the template due to abnormal gait waveforms can lead to falsely undetected strides, potentially biasing the results, as only well detected strides would be considered in subsequent analysis and interpretation of the data. This would necessitate either adjusted (disease-specific or individualized) templates or other stride segmentation methodologies. It should thus be evaluated whether severely affected gait can still be assessed using our system. Future work should focus on the validation of the system specifically for diverse groups of patients suffering from various diseases.

The results of this study may differ if a larger clinical population with a larger variety in disease severity or even other diseases had been investigated. An increased heterogeneity in the population would largely increase the external validity due to higher inter-individual variability in spatio-temporal gait parameters of patients as observed for PD, osteoarthritis or stroke [44,45,46]. The patients’ gait in this study was not severely impaired. The gait parameters were significantly different from the healthy population (i.e., significantly longer stride times and smaller steps). However, the ranges covered did not differ largely.

Slightly lower correlation coefficients between the measurement systems were observed for the gait of patients. It must be noted, though, that the number of strides contributing to the analysis is unbalanced, as only four patients contributed to the analysis and patients only represented about one-tenth of all strides performed.

Generally, test–retest reliability over the three measurement sessions was excellent. The range of ICC values as compared to other sensor-based systems was similar [11,12,13]. Only the estimation of gait velocity at slow walking was worse in comparison to other gait parameters and other studies. It has to be noted, that only five subjects of this study contributed to the estimation of the test–retest reliability, which could limit the validity of the reliability measure. The reliability results were consistent between the sensor-based and the camera-based systems, indicating that both systems could be used interchangeably for instrumented gait analysis. The sensor-based system thus presents an efficient method to acquire accurate gait parameters.

The prerequisite for the assessment of test–retest reliability is the temporal stability of gait parameters over several measurement sessions. A low reliability might be due to measurement errors or individual gait pattern changes over time. However, we observed a high reliability measure, indicating that the measurement system worked consistently well and the regarded parameters were temporally stable. The consistently lower ICCs for velocity in both measurement systems might indicate that velocity was an unstable underlying parameter.

Reliability was only assessed for healthy adults. The assumption of stability of underlying parameters renders the evaluation of retest reliability difficult in many clinical populations, as the clinical symptoms of patients suffering from gait disorders may vary over several assessment sessions. More gait data of healthy subjects and of patients should be incorporated into retest reliability assessment in future work.

Besides temporal stability of the gait patterns exhibited by the participants, the correct reattachment of the sensors to the body poses a potential error source regarding test–retest reliability. We attached the sensors using a non-removable sensor mount that was rigidly attached to the shoe. Thus, the same sensor position was assured in each retest measurement.

The laboratory like environment allowed full control over the study protocol and a supervised acquisition of gait. However, this constraint to a laboratory environment might affect gait [7] and lower the external validity of the results obtained in our study, as stride detection and the accuracy of gait parameters might be affected in unsupervised free living environments due to a higher variability of activities.

Only straight walking was evaluated in this study as the laboratory environment and the limitation of the field of view of the reference system constrained the captured movements. No turnings or other free living movements were assessed, which should be considered in future studies in order to assess the accuracy in less constrained movement situations.

The validity of the markerless motion capture system used in our study has been assessed in previous studies, where good agreement with marker-based motion analysis systems was shown. Especially, sagittal plane movements could be accurately assessed while movements in the transversal plane could not be measured equally well [47,48]. Other markerless motion capture system have also shown good agreement compared to marker-based motion capture systems [49,50], allowing the use of those systems as reference systems. A general advantage of markerless motion capture systems is that no errors due to marker misplacement occur, which is a relevant error source in marker-based motion capture systems [51,52]. Additionally to previous validation studies, the markerless video tracking system exhibited a high test–retest reliability in the assessment of the investigated spatio-temporal gait parameters in our study. Furthermore, the absence of markers allows the complementary use of both camera- and sensor- based gait assessment to enhance the quality of data in semi-supervised scenarios.

Synchronization of hardware was only assured in order to correspond the spatio-temporal parameters between the camera and the sensor system. Future work should incorporate an additional automatic synchronization between the two sensors at the feet, so that gait parameters such as double limb support and other phase information that depend on both legs can be calculated and evaluated with respect to accuracy. Additionally, other gait parameters, which could potentially yield insight into differences between healthy and pathological gait, should be implemented and assessed in the future.

Body-worn sensor systems in general still have some drawbacks as compared to stationary movement analysis systems. Obtaining kinematic information of sensor-based systems is challenging due to drift effects and inertial frame alignment [6]. Stationary systems are still considered gold standard systems, especially in biomechanical studies for the analysis of inverse kinematics and kinetics. Therefore, the most suitable movement analysis system should be chosen based on the application.

## 5. Conclusions

In summary, the sensor-based system presented in this study has great potential for the assessment of spatio-temporal gait parameters of healthy subjects and mildly affected gait of patients with PD. The possibility to quickly analyze a large number of steps that contribute to clinical decision making or treatment evaluation is an advantage compared to traditional motion capture laboratories. Given a validation in an unsupervised environment, this gait analysis system could potentially be used for unsupervised gait analysis in applications such as therapy monitoring and treatment evaluation in the future.

## Figures and Tables

**Table 1:** Population statistics for the healthy subjects and the patients with Parkinson’s disease (PD).

	Healthy Subjects	PD Patients
Gender (m:f)	6:5	2:2
Age (years)	33.6 ± 5.7	70.5 ± 6.6
Mass (kg)	77.1 ± 20.7	72.6 ± 5.3
Height (cm)	180.3 ± 9.9	172.8 ± 6.7
UPDRS-III	-	20.0 ± 6.4
Hoehn & Yahr	-	2.4 ± 0.8

**Table 2:** Overview of spatio-temporal gait parameters for eleven healthy subjects and four PD patients (*n* = 1166 steps). Shown are the mean parameters (SD), Pearson correlation coefficient *r*, bias (SD), absolute error (SD) and the relative absolute error.

Parameter	Sensor	Camera	*r*	Bias	Abs. Error	Abs. Error (%)
**Stride time (s)**	1.15 (0.18)	1.15 (0.18)	1.00	−0.000 (0.016)	0.013 (0.010)	1.1
**Stance time (s)**	0.74 (0.14)	0.70 (0.13)	0.99	0.037 (0.020)	0.037 (0.019)	5.4
**Swing time (s)**	0.41 (0.05)	0.45 (0.05)	0.93	−0.037 (0.020)	0.037 (0.019)	8.3
**Stride length (m)**	1.43 (0.22)	1.45 (0.22)	0.95	−0.014 (0.067)	0.053 (0.043)	3.6
**Velocity (m/s)**	1.30 (0.37)	1.31 (0.37)	0.99	−0.012 (0.061)	0.048 (0.040)	3.7

**Table 3:** Overview of spatio-temporal gait parameters for eleven healthy subjects and four PD patients (*n* = 1166 steps). Shown are the mean parameters (SD), Pearson correlation coefficient *r*, bias (SD), absolute error (SD) and the relative absolute error.

Parameter	Sensor	Camera	*r*	Bias	Abs. Error	Abs. Error (%)
**Stride time (s)**						
Healthy	1.13 (0.18)	1.13 (0.18)	1.00	−0.001 (0.015)	0.012 (0.009)	1.1
Patient	1.27 (0.15)	1.27 (0.15)	0.99	0.003 (0.020)	0.016 (0.013)	1.3
**Stance time (s)**						
Healthy	0.72 (0.13)	0.69 (0.13)	0.99	0.036 (0.020)	0.037 (0.019)	5.4
Patient	0.84 (0.12)	0.80 (0.12)	0.99	0.042 (0.020)	0.042 (0.020)	5.4
**Swing time (s)**						
Healthy	0.41 (0.05)	0.44 (0.05)	0.94	−0.037 (0.019)	0.037 (0.019)	8.2
Patient	0.43 (0.04)	0.47 (0.04)	0.82	−0.039 (0.026)	0.041 (0.023)	8.5
**Stride length (m)**						
Healthy	1.45 (0.21)	1.47 (0.21)	0.95	−0.016 (0.066)	0.053 (0.044)	3.6
Patient	1.25 (0.18)	1.26 (0.17)	0.93	−0.001 (0.065)	0.052 (0.039)	4.2
**Velocity (m/s)**						
Healthy	1.34 (0.37)	1.35 (0.37)	0.99	−0.013 (0.062)	0.049 (0.041)	3.6
Patient	1.01 (0.24)	1.02 (0.24)	0.98	−0.004 (0.052)	0.041 (0.031)	4.2

**Table 4:** Overview of the intraclass correlation (ICC) for the spatio-temporal gait parameters for five healthy subjects (*n* = 10 legs). Shown are the reliability of a single measurement ICC(2,1) and the reliability of the average measurement ICC(2,*k*) with k=3 repeated (test–retest) measurements.

	Sensor System		Camera System	
	ICC(2,1)	ICC(2,*k*)	ICC(2,1)	ICC(2,*k*)
**Stride time (s)**				
fast	0.89	0.96	0.91	0.97
normal	0.92	0.97	0.91	0.97
slow	0.94	0.98	0.93	0.98
**Stance time (s)**				
fast	0.87	0.95	0.89	0.96
normal	0.90	0.97	0.92	0.97
slow	0.94	0.98	0.91	0.97
**Swing time (s)**				
fast	0.92	0.97	0.83	0.94
normal	0.92	0.97	0.81	0.93
slow	0.86	0.95	0.88	0.96
**Stride length (m)**				
fast	0.87	0.95	0.87	0.95
normal	0.81	0.93	0.83	0.94
slow	0.87	0.95	0.92	0.97
**Velocity (m/s)**				
fast	0.75	0.90	0.72	0.88
normal	0.78	0.92	0.74	0.89
slow	0.55	0.79	0.55	0.79
